# Rapid corneal thinning and perforated ulcerative keratitis in a patient with relapsing polychondritis

**DOI:** 10.1186/s40662-017-0073-y

**Published:** 2017-03-16

**Authors:** Tracy Hiu Ting Lai, Nikki Far, Alvin Lerrmann Young, Vishal Jhanji

**Affiliations:** 1 Department of Ophthalmology, Tung Wah Eastern Hospital, Hong Kong, China; 2 The Chinese University of Hong Kong, Prince of Wales Hospital, Hong Kong, China; 3 UPMC Eye Center, University of Pittsburgh, 203, Lothrop Street, 15231 Pittsburgh, PA USA

**Keywords:** Relapsing polychondritis, Corneal perforation, Ulcerative keratitis

## Abstract

**Background:**

To report rapid corneal thinning and perforation in a case with relapsing polychondritis.

**Case presentation:**

A 43 year-old male diagnosed with relapsing polychondritis suffered from bilateral scleritis, bilateral swelling of pinna, saddle nose and tracheal stenosis. The patient presented with right eye pain and redness for one month. Slit lamp examination of the right eye showed 80% peripheral corneal thinning between 3 and 7 o’clock. The best-corrected visual acuity (BCVA) was 1.0 bilaterally. The degree of corneal thinning worsened to 90% after one week of oral corticosteroid use. Subsequently, topical cyclosporine 2% eye drops four times a day, oral doxycycline 100 mg twice a day and oral vitamin C 2 g daily were added. The corneal thinning gradually improved to about 60%. However, the patient rapidly tapered oral prednisolone against medical advice and returned with an acute drop in vision in his right eye. Slit lamp examination of the right eye showed peripheral corneal perforation with iris prolapse. An emergency repair with cyanoacrylate glue was performed. Intravenous methylprednisolone 1 mg/kg body weight was administered for three days and 1 g/day intravenous immunoglobulin was administered every four weeks. At 3 months postoperatively, BCVA in the right eye was 0.6. Slit lamp examination showed a well-formed anterior chamber with glue in situ.

**Conclusions:**

Relapsing polychondritis may be associated with rapid corneal thinning. The clinicians should be aware of the possibility of corneal perforation in these cases. Cyanoacrylate glue is a viable temporary management option in such scenarios.

## Background

Relapsing polychondritis is a rare systemic autoimmune condition with multiple ocular manifestations [1]. Medical treatment options include oral corticosteroids, non-steroidal anti-inflammatory drugs, immunosuppressants, biologics, and intravenous immunoglobulin [2]. We report a case of perforated peripheral ulcerative keratitis in patient with relapsing polychondritis, which required repair with cyanoacrylate glue.

## Case presentation

**Fig. 1:**
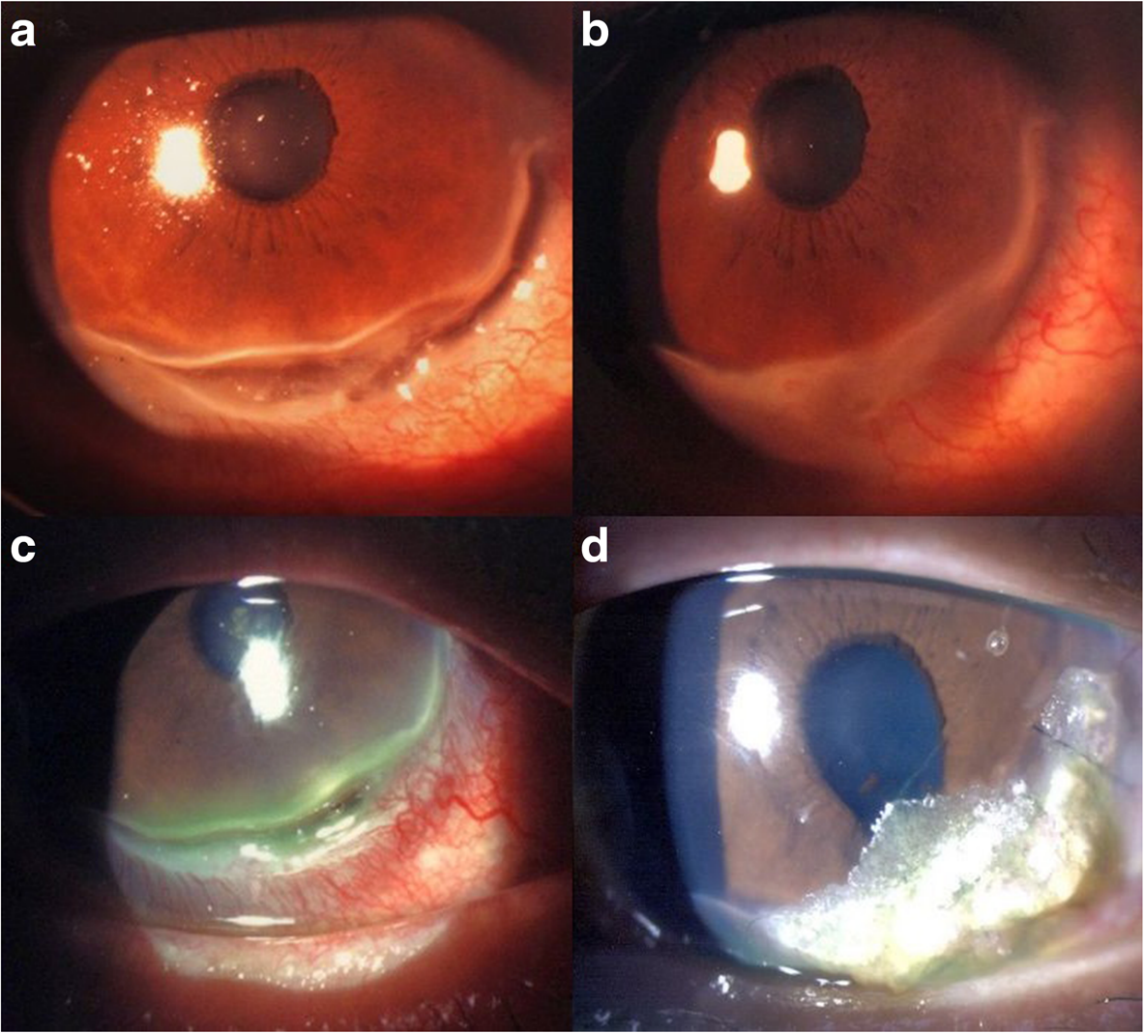
Slit lamp photographs. **a** Right eye showing 80% peripheral corneal thinning at the time of presentation; **b** Corneal thinning improved to 60% after increasing systemic corticosteroid dosage; **c** severe corneal thinning and perforation was noted after tapering of systemic corticosteroids and presented with right eye severe corneal thinning; **d** Cyanoacrylate glue applied to the area of right eye corneal perforation

A 43 year-old male diagnosed with relapsing polychondritis suffered from bilateral scleritis. He also had bilateral swelling of pinna, saddle nose and tracheal stenosis that required long-term tracheostomy. His systemic condition was resistant to azathioprine, cyclosporin A, and cyclosphamide. The patient was using topical 1% prednisolone eye drops four times a day along with preservative free ocular lubricants six times a day in both eyes. Additionally, he was on 20 mg of oral prednisolone daily and 10 mg of oral methotrexate weekly. The patient suffered from on and off bilateral scleritis that responded to topical 1% prednisolone eye drops. The patient presented with increased pain and redness in his right eye for one month. Slit lamp examination of the right eye showed 80% peripheral corneal thinning between 3 and 7 o’clock (Fig. 1a). The best-corrected visual acuity was 1.0 in both eyes. The dose of oral prednisolone was increased from 20mg to 50mg daily. However, the degree of corneal thinning progressed to 90% after one week. At this point, topical 2% cyclosporine eye drops four times a day, 100 mg of oral doxycycline twice a day and 2 g of oral vitamin C daily were added. After another week of observation, the corneal thinning gradually improved to about 60% (Fig. 1b). The dose of oral prednisolone was slowly tapered. However, the patient rapidly tapered oral prednisolone against medical advice and returned three months later with another episode of redness and pain in his right eye (Fig. 1c). The oral prednisolone was stepped up to 50 mg daily. Three days later, the patient presented to the Accident and Emergency Department of our hospital with an acute drop in visual acuity in his right eye. The right eye was markedly injected at the time of presentation. Slit lamp examination of the right eye showed corneal perforation 1.0 x 2.2 mm in size at the corneal periphery at 4 o’clock position with iris prolapse and a shallow anterior chamber. The visual acuity had dropped to 0.6. Left eye examination showed mild peripheral corneal thinning. The Seidel’s test was positive.

An emergency repair with cyanoacrylate glue was performed under local anesthesia and a bandage contact lens was placed on the cornea. Postoperatively, 0.5% levofloxacin eye drops and 1% prednisolone acetate eye drops were used four times a day. Rheumatology consult was obtained at this stage. In addition, intravenous pulse methylprednisolone 1 mg/ kg body weight was administered for three days and 1 g/day intravenous immunoglobulin was administered every four weeks with monitoring of liver and kidney function tests. At three months postoperatively, the visual acuity in the right eye was 0.6. Slit lamp examination showed a well-formed anterior chamber with glue in situ (Fig. 1d). Oral prednisolone was slowly tapered until the patient was on 20 mg of oral prednisolone daily and 1g/day of monthly intravenous immunoglobulin infusion. Unfortunately the patient passed away six months postoperatively due to respiratory failure resulting from relapsing polychondritis.

## Discussion and conclusions

Relapsing polychondritis is a rare (3.5 per million [1]) autoimmune disease characterized by episodic inflammation of cartilaginous structures in the body. The peak age of onset is 40–50 years old. There is no gender predilection. The eyes, ears, nose, joints and respiratory tract are affected. Currently, there is no specific laboratory test available for diagnosis. Ocular symptoms occur in about 65% of the patients with relapsing polychondritis [2]. These symptoms include episcleritis, scleritis, peripheral ulcerative keratitis, conjunctivitis, anterior and posterior uveitis, optic neuritis, dry eyes, cataract, extraocular muscle palsy and exophthalmos.

Peripheral ulcerative keratitis (PUK) occurs in fewer than 10% of the patients with relapsing polychondritis [3]. Bilateral destructive PUK leading to perforation, endophthalmitis, and eventually bilateral enucleation has been reported [4]. Immunologic analysis of sclera in these cases showed vasculitis and immunoglobulin deposition in vessel walls [5]. There is no standardized protocol available for the treatment of severe PUK associated with relapsing polychondritis. Topical and oral steroids are often insufficient. In refractory and destructive PUK, high-dose pulse therapy with intravenous steroids and immunosuppressants is often necessary [6]. Before starting steroids, it is important to perform corneal scraping for culture and sensitivity tests to rule out infective causes. Azathioprine, cyclosporine, cyclophosphamide and chlorambucil have been used successfully in the treatment of severe peripheral ulcerative keratitis associated with relapsing polychondritis [7, 8]. For perforated PUK, glue and patch graft can be used for repair. In our patient, the glue was in situ at three months postoperatively. Moorthy et al. studied 46 cases of herpes-related corneal perforations and found that the cyanoacrylate glue stayed in situ for an average of 39 ± 76 days (range 1–395 days) [9]. The mean duration of glue adherence was 45 days (range 2–90 days) in another case series of 22 eyes [10]. Setlik et al. showed that the cyanoacrylate glue could stay in situ for up to 270 days [11] and Tan reported a case in which the glue stayed in place for more than five years [12]. However, it is to be noted that corneal gluing is a temporizing procedure. Once the eye is quiet, a permanent surgery in terms of amniotic membrane or tectonic keratoplasty is needed.

Episcleritis and scleritis are the most common ocular manifestations of relapsing polychondritis, occurring in 47% of the patients with relapsing polychondritis [2]. Anterior scleritis is more common than posterior scleritis. It can present as diffuse, nodular or necrotizing type. In a case series with 11 relapsing polychondritis patients [5], only one patient was successfully treated with corticosteroids alone whereas seven patients required cytotoxic drugs. In particular, nodular and necrotizing scleritis was less responsive to steroids alone and required azathioprine and cyclophosphamide. Remission was achieved with infliximab in cases resistant to cytotoxic drugs [13, 14].

Current treatment of ophthalmic complications of relapsing polychondritis is largely empirical as the disease is rare with a fluctuating course. Low dose oral corticosteroids and non-steroidal anti-inflammatory drugs are used for mild cases. For moderate to severe disease with systemic involvement, high dose oral prednisolone (1mg/kg/day) and intravenous pulse methylprednisolone (1g/day) are used. In cases with severe organ involvement or no response to steroids after a few weeks, immunosuppressants are employed. In a cohort of 11 patients with active scleritis, especially for necrotizing and nodular type, 7 patients required cyclophosphamide or azathioprine [5]. The role of methotrexate in treatment of relapsing polychondritis is controversial. Whereas Letko et al. advocated the use of methotrexate for management of scleritis [15], Hoang-Xuan et al. reported two patients with relapsing polychondritis with necrotizing scleritis who did not respond to methotrexate [5]. Messmer and Foster reported that peripheral ulcerative keratitis due to relapsing polychondritis, unlike those due to rheumatoid arthritis, do not respond well to methotrexate [6].

In our case, relapsing polychondriits was resistant to many of the commonly used steroid-sparing agents. In spite of aggressive use of corticosteroids, the patient suffered from corneal perforation, which required repair with cyanoacrylate glue [16]. Although corneal gluing served as a viable interim treatment modality, the patient eventually passed away due to respiratory failure. Our case highlights the occurrence of rapid corneal thinning and corneal perforation in a resistant case of relapsing polychondritis. Clinicians should be aware of such potential complications while managing these patients.
